# Infectious Complications of Antibody–Drug Conjugates: A Review of Safety Data from FDA-Approved Agents

**DOI:** 10.1007/s11912-026-01804-4

**Published:** 2026-07-10

**Authors:** Georgios Schinas, Pantazis-Michael Voutsinas, Dimitra Stefanou, Christos Stafylidis, Aikaterini Gkoufa, Dimitrios C. Ziogas, Panagiotis T. Diamantopoulos, Helen Gogas, Amalia Anastasopoulou

**Affiliations:** First Department of Internal Medicine, School of Medicine, Laiko General Hospital, National and Kapodistrian University of Athens, 17 Agiou Thoma Street, Athens, 11527 Greece

**Keywords:** Antibody–drug conjugates, Infection, Neutropenia, Sepsis, Pharmacovigilance, Risk stratification

## Abstract

**Purpose of review:**

With fifteen agents now FDA-approved and indications expanding into earlier treatment lines, antibody–drug conjugates (ADCs) represent a rapidly growing class of targeted cancer therapeutics. Despite their selective mechanism of action, infectious complications are clinically significant and have been flagged as a disproportionate safety signal in post-marketing surveillance. This review characterizes the infection risk profiles of all fifteen FDA-approved ADCs through systematic extraction of prescribing information and pivotal trial safety data, and examines the pathophysiological mechanisms, antigen-specific clinical patterns, and evidence-based prophylaxis strategies applicable to this class.

**Recent findings:**

Calicheamicin-based agents carry the highest infection burden, with grade ≥ 3 infection rates exceeding 30–47% and near-universal severe neutropenia. CD30- and CD79b-targeting vedotin conjugates are associated with opportunistic infections—including progressive multifocal leukoencephalopathy and Pneumocystis jirovecii pneumonia—through mechanisms beyond myelosuppression, particularly T-cell immune surveillance disruption and bystander-effect lymphotoxicity. Solid tumor ADCs demonstrate lower overall infection rates with distinct organ-specific patterns: genitourinary infections predominate with Nectin-4– and Tissue Factor–directed agents, whereas pulmonary events characterize HER2- and c-Met–targeted conjugates. Linker cleavability, drug-to-antibody ratio, and payload metabolism are identified as key pharmacological determinants of myelosuppressive and infectious risk.

**Summary:**

Infectious complications of ADC therapy are clinically significant but heterogeneous, with risk profiles primarily determined by target antigen, immunologic context, and concurrent treatment. These findings support the growing adoption of a combined prevention strategy incorporating disease-based risk stratification and drug-directed infection prophylaxis.

## Introduction

Antibody-drug conjugates (ADCs) represent a sophisticated class of targeted cancer therapeutics that link tumor-associated antigen monoclonal antibodies to potent cytotoxic drugs via engineered chemical linkers [1, 2]. This concept enables selective delivery of chemotherapy to tumor cells expressing the target antigen while theoretically minimizing systemic exposure to healthy tissues [3]. The design rationale of ADCs has translated into substantial clinical benefit, with fifteen agents receiving United States Food and Drug Administration (FDA) approval across hematologic malignancies (HM) and solid tumors by 2025, and hundreds more progressing through clinical development worldwide [4, 5].

Despite their targeted intent, treatment-related adverse events with ADCs have proved both frequent and, in many instances, severe. A comprehensive meta-analysis of 169 clinical trials involving more than 22,000 patients reported that 91.2% of individuals treated with ADCs experienced at least one treatment-related adverse event, with 46.1% experiencing grade ≥ 3 events [6]. The most common high-grade toxicities reported on metanalyses on the issue, were hematologic, predominantly neutropenia affecting approximately 31% of patients and lymphopenia affecting 53% [6, 7]. These cytopenias represent the most immediate and clinically consequential drivers of infectious morbidity, predisposing patients to bacterial sepsis, invasive fungal disease, and viral reactivation syndromes [8, 9]. Reflecting the severity of these infectious complications, pneumonia (10.1%) and sepsis (7.7%) were identified among the leading causes of treatment-related death, while sepsis was also a primary driver of treatment discontinuation, accounting for 11.2% of such cases [6].

Post-marketing surveillance has confirmed severe infectious complications as safety signals. Analysis of the FDA Adverse Event Reporting System (FAERS) database spanning 2004 to 2021 identified 24,618 ADC-associated cases, with significant disproportionate reporting of sepsis-related toxicities including neutropenic sepsis (reporting odds ratio [ROR] 16.34), multiple organ dysfunction syndrome (ROR 14.77), septic shock (ROR 6.85), and bacteremia (ROR 6.49) [10]. Among reported sepsis-related cases, overall mortality approached 30%, reaching 40.1% in patients with multiple organ dysfunction syndrome [10]. Time-to-onset analysis revealed an early failure pattern, with approximately 60% of sepsis events developing within the first month of therapy and neutropenic sepsis manifesting even more rapidly, with the majority of cases occurring within three weeks of treatment initiation [10].

This temporal clustering could suggest that infection risk is front-loaded, with maximum vulnerability during the period of peak myelosuppression following initial drug exposure. Indeed, short median times-to-onset have been reported for ADC-associated hematotoxicity, ranging from 12 to 22 days across multiple agents, with hospitalization rates of 30.38% and mortality rates of 17–18% among patients experiencing these early hematologic events [11, 12]. The FAERS surveillance data also demonstrate that ADCs as a class carry a higher sepsis burden than chemotherapy alone, targeted therapy, or immunotherapy [10]. As ADC indications expand into earlier treatment lines and broader populations, characterizing their infectious complications becomes increasingly important [13].

This review synthesizes available safety data from FDA-approved prescribing information to characterize the infection risk profile of approved ADCs, examines the pathophysiological basis of ADC-associated infectious complications, identifies patterns across different ADC classes and treatment contexts, and provides data-driven insights for early recognition and prophylaxis within current guideline frameworks. Overall, this analysis aims to inform clinical decision-making and guide supportive care protocols for this expanding therapeutic class.

## Methods

### Data Sources and Search Strategy

This review analyzed safety data from ADCs approved by the FDA. Primary data were obtained from the FDA Drugs@FDA database [14], which provides comprehensive access to drug approval packages, prescribing information, and safety updates. Prescribing information was selected as the primary source because it provides standardized, FDA-reviewed safety data across all approved agents, enabling consistent cross-agent comparison. Supplementary data were obtained from pivotal clinical trial publications referenced in prescribing information, FDA clinical review documents from original approval packages, and safety supplements and label revisions. All FDA-approved ADCs with available prescribing information were eligible for inclusion. Agents were excluded only if they lacked final FDA approval or if complete prescribing information was unavailable. The search was conducted through November 2025 to capture the most recent safety information and label revisions.

### Data Extraction and Synthesis

Standardized data extraction focused on infection-related adverse events with particular attention to incidence and severity of neutropenia stratified by grade using Common Terminology Criteria for Adverse Events [15], rates of febrile neutropenia (FN), specific infection types and anatomic sites, grade ≥ 3 infectious complications, fatal infectious events, documented opportunistic infections (OI), and boxed warnings related to infectious complications. Data were extracted from both monotherapy and combination therapy studies when available, with specific notation of treatment regimens and comparator arms. For combination therapies, safety data were analyzed in the context of the specific regimen studied, acknowledging that combination effects may confound attribution to the ADC component. Post-marketing pharmacovigilance data from the FDA FAERS database were incorporated from published analyses to provide real-world signal context.

## Results

### Overview of Included Agents

Safety data were extracted for 15 FDA-approved ADCs spanning HM (targeting CD30, CD79b, CD22, CD33, CD19, and BCMA) and solid tumors (targeting HER2, Trop-2, Nectin-4, Tissue Factor, FRα, and c-Met) (Table 1). The analysis encompassed pivotal phase 2 and phase 3 trials with populations ranging from 57 to 1,486 patients per study arm.

**Table 1:** Infection-related safety data of FDA-approved antibody–drug conjugates

ADC (Brand Name)	Indication / Population	Trial (NCT ID)	Treatment Arm(s)	*N*	Grade ≥ 3 Neutropenia / FN	Grade ≥ 3 Infections (Types / Fatal Events)	Opportunistic Infections / Special Warnings
CD33 (AML)							
Gemtuzumab ozogamicin [16]	Newly diagnosed CD33 + AML (with DA)	ALFA-0701 (NCT01350947)	GO + DA (*n* = 131) vs. DA alone (*n* = 137)	271	Near-universal grade ≥ 3–4 (~ 100%)	Grade ≥ 3 infection: Induction 47% vs. 39%; Consolidation 1: 55% vs. 42%; Consolidation 2: 50% vs. 50%. Fatal infection: 3% vs. 1%. Postmarketing: PJP, invasive fungal pneumonia, *Stenotrophomonas* infections	Boxed warning: hepatotoxicity (VOD/SOS). PJP, invasive pulmonary mycosis, neutropenic colitis (incl. fatal)
*(continued)*	Elderly/unfit newly diagnosed AML	AML-19	GO monotherapy (*n* = 118) vs. BSC (*n* = 119)	237	Frequent grade ≥ 3; FN: 18% vs. 24%	Grade ≥ 3 infection: 35% vs. 34%. Any-grade infection: 44%	Same as above
*(continued)*	Pediatric/young adult AML	AAML0531 (NCT00372593)	GO + chemotherapy (*n* = 520) vs. chemotherapy alone (*n* = 517)	1,037	FN: 32% vs. 30% (Induction 1); 24% vs. 22% (Intensification 2)	Grade ≥ 3 infection: 36% vs. 35% (Induction 1); 67% vs. 69% (Intensification 2). Fatal infection: 3% vs. 1%	Same as above
*(continued)*	R/R AML	MyloFrance-1	GO monotherapy (single arm)	57	High-grade neutropenia; not fully quantified	Any-grade infection: 42%. Grade 3 sepsis: 32%; Grade 3 pneumonia: 7%	Same as above
*CD22 (ALL*,* HCL)*							
Inotuzumab ozogamicin [17]	R/R B-cell precursor ALL	INO-VATE ALL (NCT01564784)	InO (*n* = 164) vs. investigator’s choice chemotherapy (*n* = 143)	307	Grade 3–4: 86% vs. 88%. FN: 26% vs. 53%	Grade ≥ 3 infection: 28% vs. 54%. Infections in 79/165 patients (48%); sepsis/bacteraemia 17%, lower RTI 12%, upper RTI 12%, fungal 9%, viral 7%, GI 4%, skin 4%, bacterial 1%. Fatal infections: 5% (8/164), incl. fatal pneumonia, neutropenic sepsis, pseudomonal sepsis, septic shock	Boxed warning: hepatotoxicity (VOD/SOS), increased post-HSCT non-relapse mortality. HBV/VZV reactivation (rare)
Moxetumomab pasudotox [18]	R/R hairy cell leukemia (≥ 2 prior therapies incl. PNA)	Study 1053 (NCT01829711)	Moxetumomab monotherapy (single arm)	80	Grade 3: 11%; Grade 4: 20%	Not separately quantified at grade ≥ 3	Boxed warning: capillary leak syndrome and HUS
***CD30 (HL***,* ALCL*,* PTCL)*							
Brentuximab vedotin [19]	R/R HL post-ASCT	SG035-0003 (NCT00848926)	BV monotherapy (single arm)	102	Grade 3: 15%; Grade 4: 6%	Grade 3–4 infection: low. Pneumonia: 1%. PJP: 1%. Pulmonary aspergillosis: 1%. MRSA UTI: 1%. Pyelonephritis: 2%	Boxed warning: PML (fatal cases; onset within 3 months)
*(continued)*	R/R sALCL	SG035-0004 (NCT00866047)	BV monotherapy (single arm)	58	Grade 3: 12%; Grade 4: 9%	UTI: 3%. Septic shock: 3%	PML (fatal case reported)
*(continued)*	Untreated stage III/IV cHL	ECHELON-1 (NCT01712490)	BV + AVD (*n* = 662) vs. ABVD (*n* = 659)	1,334	BV + AVD: Grade 3: 20%, Grade 4: 62%; FN: 19%. ABVD: Grade 3: 31%, Grade 4: 42%; FN: 8%	Pneumonia: 3%	Boxed warning: PML
*(continued)*	cHL post-auto-HSCT consolidation	AETHERA (NCT01100502)	BV (*n* = 167) vs. placebo (*n* = 160)	329	BV: Grade 3: 30%, Grade 4: 9%. Placebo: Grade 3: 6%, Grade 4: 4%	No grade 3–4 URTI in BV arm vs. 1% in placebo	—
*(continued)*	Untreated HL in children/young adults	AHOD1331 (NCT02166463)	BV + AVEPC (*n* = 300) vs. ABVE-PC (*n* = 300)	600	BV+AVEPC: Grade 3: 8%, Grade 4: 43%; FN: 31%. ABVE-PC: Grade 3: 4%, Grade 4: 36%; FN: 33%	Grade ≥ 3 infection: 12% vs. 10%. Sepsis: 2.7% vs. 4.2%	—
*(continued)*	Untreated sALCL/CD30 + PTCL	ECHELON-2 (NCT01777152)	BV + CHP (*n* = 223) vs. CHOP (*n* = 226)	449	BV + CHP: Grade 3/4: 39%. CHOP: 36%. FN: 19% vs. 16%	Pneumonia: 5%. Sepsis: 3%	—
*(continued)*	R/R LBCL	L-MIND extension (randomized)	BV + lenalidomide + rituximab (*n* = 112) vs. placebo + Len + R (*n* = 118)	230	Grade 3–4: 49% vs. 42%. FN: 7%	Pneumonia (incl. PJP, fungal, viral): 21% vs. 9%. COVID-19: 13% vs. 8%. Sepsis: 9%. UTI: 3.6%	—
***CD79b (DLBCL)***							
Polatuzumab vedotin [20]	R/R DLBCL (≥ 2 prior lines, with BR)	GO29365 (NCT02257567)	Pola + BR (*n* = 45) vs. BR (*n* = 39); expanded safety (*n* = 173)	84 (pivotal); 173 (expanded)	Pola + BR: 61% vs. BR: 33%. FN: 11–13%	Grade ≥ 3 infection: 32% (expanded). Infection-related death: 2.9%. Pneumonia: 16% vs. 2.6% (incl. PJP, fungal). Sepsis: 6–7% (4 fatalities). CMV; Herpesvirus	PML: 0.6% (1/173). PJP/herpesvirus prophylaxis recommended
*(continued)*	Untreated DLBCL/HGBL (IPI ≥ 2, with R-CHP)	POLARIX (NCT03274492)	Pola + R-CHP (*n* = 435) vs. R-CHOP (*n* = 438)	873	Pola + R-CHP: 39% vs. R-CHOP: 42%	Grade 3–4 infection: 14%. Infection-related death: 1.1%. Fatal pneumonia: 0.9%. Fatal sepsis: 0.2%. CMV; herpesvirus	CMV and herpesvirus infection reported
*CD19 (LBCL)*							
Loncastuximab tesirine [21]	R/R LBCL (≥ 2 prior systemic lines)	LOTIS-2 (NCT03589469)	Monotherapy (single arm)	145	All grades: 52%; Grade 3–4: 30%; FN: 3%	URTI: <1% grade 3–4. Pneumonia: 5% (all grades). Sepsis: 2%. Fatal infection: 1%	—
*BCMA (Multiple Myeloma)*							
Belantamab mafodotin [22]	R/R multiple myeloma [≥ 2 prior lines, with Bd]	DREAMM-7 (NCT04246047)	Belamaf + Bd (*n* = 242) vs. daratumumab + Bd (*n* = 246)	488	Grade 3–4: 17% vs. 13%	Pneumonia: 16% vs. 5% (10 vs. 7 fatal). COVID-19: 5% vs. 2% (3 vs. 5 fatal). Sepsis: 4% (2 fatal). URTI: 2% (1 fatal)	Ocular toxicity (corneal events, keratopathy, corneal ulcers)
*HER2 (Breast Cancer)*							
Trastuzumab deruxtecan [23]	HER2 + metastatic BC (≥ 2 prior anti-HER2)	DESTINY-Breast01 + J101	T-DXd monotherapy (single arm, combined safety)	234	Grade 3–4: 16%; FN: 1.7%	URTI: no grade 3–4 (all grades 15%). Fatal pneumonia: 0.4%	Boxed warning: ILD/pneumonitis
*(continued)*	HER2 + metastatic BC (prior anti-HER2)	DESTINY-Breast03 (NCT03529110)	T-DXd (*n* = 257) vs. T-DM1 (*n* = 261)	518	T-DXd: 18% vs. T-DM1: 2.3%	Respiratory infection: 0.8%. Fatal COVID-19: 0.4%	Same as above
*(continued)*	HER2 + metastatic BC	DESTINY-Breast02	T-DXd (*n* = 404) vs. TPC (*n* = 195)	599	T-DXd: 16% vs. TPC: 4.7%	Fatal pneumonia: 0.2%. Fatal hepatitis B: 0.2%. Fatal COVID-19: 0.2%	Same as above
*(continued)*	HR+/HER2-low metastatic BC	DESTINY-Breast04 (NCT03734029)	T-DXd (*n* = 371) vs. physician’s choice chemotherapy (*n* = 172)	543	T-DXd: 14% vs. Chemo: 38%; FN: 0.3% (1 fatal)	URTI: 0.3% vs. 0%. Fatal sepsis: 0.5% (2/371)	Same as above
*(continued)*	HR+/HER2-low or ultralow metastatic BC	DESTINY-Breast06 (NCT04494425)	T-DXd (*n* = 434) vs. chemotherapy (*n* = 417)	851	Grade 3–4:27% vs. 20% FN: 1.2% (T-DXd)	URTI: no grade 3–4 (all grades 19% vs. 9%). COVID-19/pneumonia: 0.9% (0.2% fatal). Sepsis: 0.5%. Neutropenic sepsis: 0.2%. Bacterial meningoencephalitis: 0.2%	Same as above
*(continued)*	Various solid tumors (combined safety)	DB01/DPT02/DL01/DCR02	T-DXd monotherapy (combined safety population)	347	Grade 3–4: 21%	Pneumonia: 2.3%. Fatal sepsis: 0.6%. Fatal COVID-19: 0.6%. Cellulitis	Same as above
Ado-trastuzumab emtansine [24]	HER2 + metastatic BC (post-trastuzumab + taxane)	EMILIA (NCT00829166)	T-DM1 (*n* = 490) vs. lapatinib + capecitabine (*n* = 488)	991	Grade 3–4: 2% vs. 4.3%	Grade 3–4 UTI: 0.6% vs. 0%	Boxed warning: hepatotoxicity, cardiac toxicity, embryo-fetal toxicity
*(continued)*	HER2 + early BC adjuvant (residual disease)	KATHERINE (NCT01772472)	T-DM1 (*n* = 740) vs. trastuzumab (*n* = 720)	1,486	T-DM1: Grade 3: 1%, Grade 4: 0% vs. Tras: Grade 3: 0.6%, Grade 4: 0.6%	UTI: 0.3% vs. 0.1%	—
*Nectin-4 (Urothelial Cancer)*							
Enfortumab vedotin [25]	LA/M urothelial cancer (post-PD-1/L1 + platinum)	EV-201 Cohort 1 (NCT03219333)	EV monotherapy (single arm)	125	Grade 3–4: 5%; FN: 4%	UTI: 6%. Sepsis: 3%. Fatal aspiration pneumonia: 0.8%. Fatal sepsis: 0.8%. Herpes zoster: 3%. Cellulitis: 5%	Herpes zoster reactivation: 3%
*(continued)*	LA/M urothelial cancer (previously untreated, with pembrolizumab)	EV-302 (NCT04223856)	EV + pembrolizumab (*n* = 440) vs. gemcitabine + platinum (*n* = 433)	873	EV+pembro: Grade 3–4: 9% vs. Chemo: 50%	Grade 3–4 UTI: 5% vs. 8%. Pneumonia: 2.3%. Fatal pneumonia: 0.5%	—
*(continued)*	LA/M urothelial cancer (previously untreated, cisplatin-ineligible)	EV-103 (NCT03288545)	EV + pembrolizumab (multi-cohort, single arm)	121	Grade 3–4: 12%	Grade 3–4 UTI: 12%. Urosepsis: 5%. Sepsis: 3.3%. Pneumonia: 3.3%. Fatal sepsis: 1.6%	—
*(continued)*	LA/M urothelial cancer (post-PD-1/L1 + platinum)	EV-301 (NCT03474107)	EV (*n* = 296) vs. chemotherapy (*n* = 291)	608	Grade 3–4: 12% vs. 17%	Grade 3–4 UTI: 6% vs. 3%. Pneumonia: 5%. Fatal septic shock: 0.3%. Fatal pelvic abscess: 0.3%	—
*(continued)*	LA/M urothelial cancer (post-PD-1/L1, cisplatin-ineligible)	EV-201 Cohort 2 (NCT03219333)	EV monotherapy (single arm)	89	Grade 3–4: 9%	Pneumonia: 5%. Fatal pneumonia: 1.1%. Sepsis: 5%. Fatal sepsis: 1.1%	—
*Tissue Factor (Cervical Cancer)*							
Tisotumab vedotin [26]	R/M cervical cancer (post-chemotherapy)	innovaTV 204 (NCT03438396)	TV monotherapy (single arm)	101	Grade 3–4: 3%	UTI/cystitis: 2%. Pneumonia: 4%. Sepsis: 3%. Septic shock: 1%	Ulcerative keratitis
*(continued)*	R/M cervical cancer	innovaTV 301 (NCT04697628)	TV (*n* = 250) vs. chemotherapy (*n* = 239)	489	Grade 3–4: 3.6% vs. 13.4%	UTI: 4.4% vs. 7.1%. Pneumonia: 0.4% vs. 0.4%. Sepsis: 2% vs. 0.8% (1 fatal). COVID-19: 2% vs. 1.7%. Fatal pneumonia: 0.4%	Same as above
*Trop-2 (Breast Cancer*,* NSCLC)*							
Sacituzumab govitecan [27]	mTNBC (≥ 2 prior lines for metastatic disease)	IMMU-132-01 (NCT01631552)	SG monotherapy (single arm)	108	Grade 3–4: 43% (Grade 4: 26% in UGT1A1*28 homozygous). FN: 8%	Respiratory tract infection: 3%. Pneumonia: 2%. UTI: 3%. Neutropenic colitis: 2%	Boxed warning: severe neutropenia and severe diarrhea. Neutropenic colitis: 2%
*(continued)*	mTNBC (post-taxane + ≥ 2 chemo)	ASCENT (NCT02574455)	SG (*n* = 258) vs. single-agent chemotherapy (*n* = 224)	482	Grade 3–4: 49% vs. 36%	URTI: no grade 3–4. Pneumonia: 3%. Fatal pneumonia: 0.4%. UTI: 0.4% (both arms)	—
*(continued)*	HR+/HER2 − metastatic BC (post-CDK4/6i + endocrine therapy + taxane)	TROPiCS-02 (NCT03901339)	SG (*n* = 268) vs. single-agent chemotherapy (*n* = 249)	517	Grade 3–4: 53% vs. 40%; FN: 4%	Pneumonia: 2%. Fatal COVID-19: 0.4%. Fatal septic shock: 0.4%. Neutropenic colitis: 2%	Neutropenic colitis: 2%; colitis: 2%
*(continued)*	UGT1A1 genotype analysis (combined safety)	Combined safety population	SG monotherapy (combined, *N* = 948)	948	Grade 3–4: 49%. *28 homozygous: 58%; heterozygous: 49%; wild-type: 43%. FN: 6%	Neutropenic colitis: 1.4%	UGT1A1*28 homozygosity increases neutropenia severity
Datopotamab deruxtecan [28]	HR+/HER2 − metastatic BC (post-endocrine therapy+ chemo)	TROPION-Breast01 (NCT05104866)	Dato-DXd (*n* = 360) vs. chemotherapy (*n* = 351)	711	Grade 3–4: 1.6% vs. 35%	UTI: 1.9%. COVID-19: 1.4% vs. 0.9%	Oral mucositis, stomatitis, keratitis, blepharitis
*(continued)*	EGFR-mutated NSCLC (combined safety)	TROPION-Lung05/01/PanTumor01	Dato-DXd monotherapy (combined safety population)	125	Not separately reported	Grade 3–4 COVID-19: 2.4%	Stomatitis: 71% any grade; 9% grade 3–4
*c-Met (NSCLC)*							
Telisotuzumab vedotin [29]	LA/M non-squamous NSCLC (c-Met high)	LUMINOSITY (NCT03539536)	Teliso-V monotherapy (single arm)	168	Grade 3–4: 1.2%	Pneumonia (grouped, incl. COVID-19): 6%. Fatal pneumonia: 1.2% (2/168)	Keratitis: 11%. Non-infectious endocarditis: 0.6%
*Folate Receptor α (Ovarian Cancer)*							
Mirvetuximab soravtansine [30]	FRα + platinum-resistant ovarian/FT/peritoneal cancer	Study 0417 (NCT04296890)	Mirvetuximab monotherapy (single arm)	106	All grades: 26%; Grade 3–4: 3%	Grade ≥ 3 infection: 3%	Boxed warning: ocular toxicity
*(continued)*	FRα + platinum-resistant ovarian/FT/peritoneal cancer	MIRASOL (NCT04209855)	Mirvetuximab (*n* = 218) vs. chemotherapy (*n* = 207)	453	Mirv: Grade 3–4: 1% vs. Chemo: 17%	Fatal neutropenic sepsis reported	Same as above

Abbreviations: *ADC* antibody–drug conjugate, *ABVD* doxorubicin/bleomycin/vinblastine/dacarbazine, *ALL* acute lymphoblastic leukemia, *AML* acute myeloid leukemia, *ASCT* autologous stem cell transplantation, *AVD* doxorubicin/vinblastine/dacarbazine, *BC* breast cancer, *Bd* bortezomib/dexamethasone, *BR* bendamustine/rituximab, *BSC* best supportive care, *BV* brentuximab vedotin, *CDK4/6i* cyclin-dependent kinase 4/6 inhibitor, *cHL* classical Hodgkin lymphoma, *CHP* cyclophosphamide/doxorubicin/prednisone, *CHOP* cyclophosphamide/doxorubicin/vincristine/prednisone, *CMV* cytomegalovirus, *DA* daunorubicin/cytarabine, *DLBCL* diffuse large B-cell lymphoma, *EV* enfortumab vedotin, *FN* febrile neutropenia, *FRα* folate receptor alpha, *FT* fallopian tube, *GO* gemtuzumab ozogamicin, *HCL* hairy cell leukemia, *HGBL* high-grade B-cell lymphoma, *HL* Hodgkin lymphoma, *HSCT* hematopoietic stem cell transplantation, *HUS* hemolytic uremic syndrome, *ILD* interstitial lung disease, *InO* inotuzumab ozogamicin, *IPI* International Prognostic Index, *LA/M* locally advanced or metastatic, *LBCL* large B-cell lymphoma, *MRSA* methicillin-resistant Staphylococcus aureus, *NSCLC* non-small cell lung cancer, *PJP* Pneumocystis jirovecii pneumonia, *PML* progressive multifocal leukoencephalopathy, *PNA* purine nucleoside analog, *PTCL* peripheral T-cell lymphoma, *R-CHP* rituximab/CHP, *R-CHOP* rituximab/CHOP, *R/M* recurrent or metastatic, *R/R* relapsed or refractory, *sALCL* systemic anaplastic large cell lymphoma, *SG* sacituzumab govitecan, *T-DM1* ado-trastuzumab emtansine, *T-DXd* trastuzumab deruxtecan, *TPC* treatment of physician’s choice, *TV* tisotumab vedotin, *UTI* urinary tract infection, *VOD/SOS* veno-occlusive disease/sinusoidal obstruction syndrome, *VZV* varicella-zoster virus

Data extracted from FDA-approved prescribing information and pivotal clinical trial publications. Agents are organized by target antigen and grouped into hematologic and solid tumor indications. Infection rates represent grade ≥ 3 events unless otherwise specified. Treatment arm details include sample sizes per arm where available from randomized trials. Comparator arm data are provided where available from randomized trials (format: ADC arm vs. comparator arm). Single-arm studies are indicated as “single arm.” “—” indicates no notable opportunistic infections or special warnings beyond those listed. When multiple trials exist for an agent, subsequent rows are marked “(continued)” in the ADC column. Fatal events are explicitly noted. Neutropenia and infection rates from FDA labels may differ slightly from primary publications due to different safety populations or reporting conventions

### Infection Profiles of ADCs Employed in Hematologic Malignancies

#### Calicheamicin-Based ADCs in Acute Leukemias

Agents utilizing calicheamicin payloads exhibited the highest infection burden among all approved ADCs. In the ALFA-0701 trial involving patients with acute myeloblastic leukemia (AML), grade ≥ 3 infections occurred in 47% of GO recipients versus 39% of controls during induction, with persistent elevations during consolidation (55% vs. 42% in the first cycle and 50% vs. 50% in the second) [31]. In the AML-19 monotherapy study, FN was observed in 18% of GO-treated patients versus 24% in the best supportive care arm [32]. Fatal infections occurred in 3% of GO-treated patients versus 1% in the chemotherapy-alone arm of AAML0531 [33]. In the MyloFrance-1 monotherapy study, any-grade infection reached 42%, with grade 3 sepsis in 32% and grade 3 pneumonia in 7% [34]. Post-marketing surveillance has further identified *Pneumocystis jirovecii* pneumonia (PJP), invasive pulmonary mycosis, *Stenotrophomonas maltophilia* infections, and neutropenic colitis, including fatal cases [16].

InO in relapsed/refractory (R/R) acute lymphoblastic leukemia (ALL) was associated with FN in 26% of patients versus 53% in the chemotherapy arm, whereas grade ≥ 3 infections occurred in 28% and 54%, respectively [17]. Fatal infections accounted for 5% of the InO population, encompassing pneumonia, neutropenic sepsis, pseudomonal sepsis, and septic shock.

#### Vedotin-Based ADCs in Lymphomas

Brentuximab vedotin (BV) demonstrated marked variability in infectious risk depending on treatment context. As monotherapy in R/R Hodgkin lymphoma (HL) in SG035-0003 trial, grade 3 infections - including PJP, pulmonary aspergillosis, and methicillin-resistant *Staphylococcus aureus* (MRSA) urinary tract infection (UTI)- each occurred in approximately 1% of patients [35]. In R/R systemic anaplastic large-cell lymphoma (sALCL) (SG035-0004 trial), septic shock was reported in 3% [36]. BV carries a Boxed Warning for progressive multifocal leukoencephalopathy (PML), with fatal cases confirmed across HL and sALCL cohorts, occurring within 3 months of initial exposure [19].

In combination regimens, infectious risk escalated substantially. In ECHELON-1, BV plus AVD produced FN in 19%, compared to 8% FN with ABVD [37]. In the pediatric AHOD1331 trial, FN occurred in 31% of the BV-containing arm versus 33% in the comparator, with grade ≥ 3 infections in 12% versus 10% and sepsis in 2.7% versus 4.2% [38]. The ECHELON-2 trial in untreated peripheral T-cell lymphoma (PTCL) demonstrated pneumonia (5%) and sepsis (3%) in the experimental arm [39].

Polatuzumab vedotin (Pola) in combination with bendamustine-rituximab (BR) for R/R diffuse large B-cell lymphoma (DLBCL) (GO29365) produced FN in 11–13% [40]. In the expanded safety population, grade ≥ 3 infections reached 32%, with infection-related mortality of 2.9% within 90 days. Pneumonia occurred in 16% of Pola recipients versus 2.6% in controls, encompassing PJP and fungal etiologies, while sepsis occurred in 6–7% with four fatalities. Herpesvirus infections, including Cytomegalovirus (CMV), were also documented, prompting a label recommendation for PJP and herpesvirus prophylaxis [20]. A single case of PML (0.6%) was reported in the expanded cohort. In the frontline POLARIX trial, grade 3–4 infections occurred in 14% of the Pola plus R-CHP arm, with infection-related deaths in 1.1% [41].

Loncastuximab tesirine (Lonca), carrying a pyrrolobenzodiazepine dimer payload, induced pneumonia (5% all grades), sepsis (2%), and fatal infection in 1% [21].

#### Other Hematologic ADCs

Belantamab mafodotin (BelaM) [22], utilizing an MMAF payload in R/R multiple myeloma (MM), was associated with a distinctive respiratory signal in DREAMM-7 [42]. Pneumonia occurred in 16% of BelaM-treated patients versus 5% in the daratumumab-bortezomib-dexamethasone (DVd) comparator, including 10 versus 7 fatal cases. COVID-19 was reported in 5% versus 2% (3 vs. 5 fatal), and sepsis in 4% (2 fatal). Moxetumomab pasudotox, a *Pseudomonas* exotoxin–based conjugate for R/R hairy cell leukemia, carries a Boxed Warning for capillary leak syndrome and hemolytic uremic syndrome rather than infectious complications per se [18].

### Infection Profiles in ADCs Employed in Solid Tumors

#### HER2-Targeted ADCs in Breast Cancer

The DESTINY-Breast03 trial provided direct comparative data between trastuzumab deruxtecan (T-Dxd) and ado-trastuzumab emtansine (T-DM1) [43]. Grade ≥ 3 infectious events were infrequent in both arms, with respiratory infection in 0.8% and fatal COVID-19 in 0.4% of T-DXd–treated patients [44]. Across the broader DESTINY program, FN remained generally below 2%. Infectious complications across studies also included pneumonia (2.3% in the combined safety population), fatal sepsis (0.5–0.6%), fatal COVID-19, fatal hepatitis B reactivation (HBVr), and a rare case of bacterial meningoencephalitis. Interstitial lung disease (ILD)/pneumonitis represents the primary Boxed Warning for T-Dxd ([23].

Regarding T-DM1, infectious events were limited to low-grade UTIs (grade 3–4 events: 0.3–0.6%) [24].

#### Trop-2–Targeted ADCs

Sacituzumab govitecan (SG) was associated with FN in 4–8% of patients (44). Infectious complications included grade ≥ 3 pneumonia (2–3%), respiratory tract infections (3%), and UTIs (3%) in the IMMU-132-01 study, whereas neutropenic colitis occurred in 1.4–2% across trials. The TROPiCS-02 trial documented fatal COVID-19 and fatal septic shock, each in 0.4% of the treatment population [45]. SG carries a Boxed Warning for severe neutropenia and severe diarrhea [27].

Datopotamab deruxtecan (Dato-Dxd) demonstrated grade ≥ 3 infections which were limited to UTIs (1.9%) and COVID-19 (1.4%) (42). However, a high incidence of stomatitis (71% any grade; 9% grade 3–4 in the NSCLC combined safety population) was noted [28].

#### Vedotin-Based ADCs in Genitourinary and Cervical Cancers

Enfortumab vedotin (EV) in urothelial cancer was associated with grade 3–4 UTIs in 5–12% of patients across multiple cohorts (EV-201, EV-301, EV-302, EV-103), with urosepsis in 5% of the cisplatin-ineligible monotherapy population [25]. Fatal events included sepsis, septic shock, aspiration pneumonia, and pelvic abscess [25]. Herpes zoster reactivation was documented in 3% of the EV-201 Cohort 1 population [46].

Tisotumab vedotin in cervical cancer demonstrated clinically significant infections including UTI (2–4.4%), pneumonia (0.4–4%), sepsis (2–3%), and septic shock (1%) across innovaTV 204 and innovaTV 301 [26]. Fatal sepsis and fatal pneumonia were each reported in < 1%.

#### Emerging ADCs in Solid Tumors

Telisotuzumab vedotin in c-Met–overexpressing NSCLC (LUMINOSITY) was associated with grade ≥ 3 pneumonia in 6% of patients, including fatal pneumonia in 1.2% (2/168), however, trial conduct during the COVID-19 pandemic with inclusion of COVID-19 pneumonia in this percentage complicates interpretation of ADC-attributable risk [29].

Mirvetuximab soravtansine in FRα-positive platinum-resistant ovarian cancer demonstrated low infection rates (grade ≥ 3 infection: 3% in Study 0417), although fatal neutropenic sepsis was reported in the MIRASOL trial [47]. The primary safety concern is ocular toxicity (Boxed Warning) rather than infectious complications [30].

## Discussion

### Neutropenia as the Primary Determinant of Infection Risk

The spectrum of neutropenia varies dramatically across approved agents. Maximum rates occur with hematologic indications: GO causes near-universal severe neutropenia, InO reaches 86% grade ≥ 3 neutropenia, Pola plus BR 61% and BV plus AVD 82% combined grade 3–4 neutropenia [48–50]. SG produced the highest neutropenia rates among solid tumor ADCs, and occupies an intermediate position at approximately 50%, with pharmacogenomic modulation based on UGT1A1 status [51, 45]. In contrast, certain solid tumor ADCs demonstrated remarkably low myelosuppression: T-DM1 at 2.3% and Dato-Dxd at 1.1% [52, 53].

The data from this review affirms that neutropenia remains the predominant driver of infection risk across the ADC class. Calicheamicin-based ADCs rank among the highest for grade ≥ 3 infection rates. At the opposite extreme, T-DM1’s minimal myelosuppression translates into negligible infectious complications, with UTIs occurring in less than 1% of patients. Between these poles, the MMAE-vedotin conjugates for HM occupy graded intermediate positions, with infection rates tracking their myelosuppressive potential.

The pharmacovigilance analysis by Xia et al. provides independent validation of this neutropenia-driven risk paradigm. Calicheamicin-based agents exhibited the highest FAERS sepsis reporting odds ratios—GO (ROR 28.52; 95% CI 26.80–30.35) and InO (ROR 10.53; 95% CI 9.22–12.01)—ranking first and second among all evaluated agents [10]. The MMAE-vedotin class clustered within a narrow intermediate range (ROR 7.33–8.30), while T-DM1 exhibited the lowest signal (ROR 2.65).

### Mechanistic Basis of ADC Neutropenia

The spectrum of neutropenia among currently available ADCs is driven not only by therapeutic context—ADCs used in combination with intensive chemotherapy inevitably show higher rates than monotherapy—but also by intrinsic drug properties. GO targets CD33-expressing cells of the myeloid lineage, and neutropenia consequently reflects an on-target effect. On the contrary, neutropenia associated with most other ADCs arises through target-independent mechanisms, predominantly related to extracellular release of their cytotoxic payloads, highlighting off-target toxicity [54]. Three inter-related pharmacological factors seem to account for the wide off-target neutropenia spectrum, namely linker type, drug-to-antibody ratio (DAR), and payload metabolism [54].

The chemical linker connecting the target-specific, antibody part of the ADC with the cytotoxic payload, is the decisive determinant of systemic payload exposure. Cleavable linkers, such as the hydrazone acid-labile linkers (GO) and the peptide-based cathepsin-sensitive linkers (vedotin conjugates), are inherently less stable in circulation, as conditions such as acidic pH in normal tissues can trigger premature payload release [55]. This instability increases systemic exposure to the free cytotoxic agent, driving off-target myelotoxicity and bystander killing of antigen-negative cells [56]. Non-cleavable linkers, such as the thioether linkage in T-DM1, require full antibody internalization and lysosomal degradation before payload release, conferring markedly greater plasma stability [57]. Clinical data confirm this distinction: in pooled analysis, grade ≥ 3 adverse events were significantly more frequent with cleavable versus non-cleavable linkers (weighted risk difference − 12.9%; 95% CI − 17.1% to − 8.8%) [58]. Notably, neutropenia accounted for the greatest absolute difference of favoring non-cleavable constructs among ≥ grade 3 toxicities (− 9.1%; 95% CI − 12% to − 6.2%). A specific mechanism involving linker stability has been postulated: serine proteases, particularly elastase, secreted by differentiating neutrophils in the bone marrow, cleave valine-citrulline linkers extracellularly, liberating free payload that destroys neighboring hematopoietic precursors in a self-amplifying cycle [59]. Non-cleavable linkers were resistant to this proteolytic process, consistent with the substantially lower neutropenia rates observed with agents such as T-DM1.

The DAR further modulates off-target toxicity risk. Approved ADCs span a DAR range of 2–8; despite greater in vitro potency, higher DAR accelerates plasma clearance without proportional efficacy gains, explaining the enduring pharmacokinetic preference for DAR = 4 constructs [60, 61]. In a recent retrospective analysis of ADC clinical trials, a DAR exceeding 4 was independently associated with an 18.3-fold increase in the odds of hematologic toxicity [62]. The two Trop-2–directed ADCs provide a pharmacological case study on how DAR affects neutropenia risk. SG’s DAR of approximately 7.6 [63], which is the highest among approved ADCs, is feasible because its payload, SN-38, is moderately potent rather than ultra-cytotoxic, but the trade-off is substantially greater systemic cytotoxic exposure and consequently more pronounced myelosuppression. Dato-Dxd employs a lower DAR of 4, achieving efficacy through targeted intracellular delivery of DXd, a payload more than 10-fold more potent than SN-38, rather than systemic exposure [64]. The result is a toxicity profile dominated by mucosal effects (stomatitis 71% any grade) rather than myelosuppression.

Finally, payload metabolism independently modulates neutropenia severity beyond linker chemistry or DAR. SN-38, the active metabolite of irinotecan and the cytotoxic warhead of SG, undergoes hepatic glucuronidation via UGT1A1, and impaired clearance in patients homozygous for the UGT1A12*8 polymorphism substantially amplifies toxicity specific to this agent. A meta-analysis of four SG trials encompassing 999 genotyped patients demonstrated that UGT1A12*8 homozygosity was associated with an over sevenfold increase in the odds of combined severe grade ≥ 3 toxicity (OR 7.03; 95% CI 3.41–14.50), including significantly elevated risks of neutropenia (OR 1.80; 95% CI 1.03–3.14) alongside higher rates of dose reduction and treatment interruption [65].

### Mechanisms Beyond Neutropenia

**Fig. 1:**
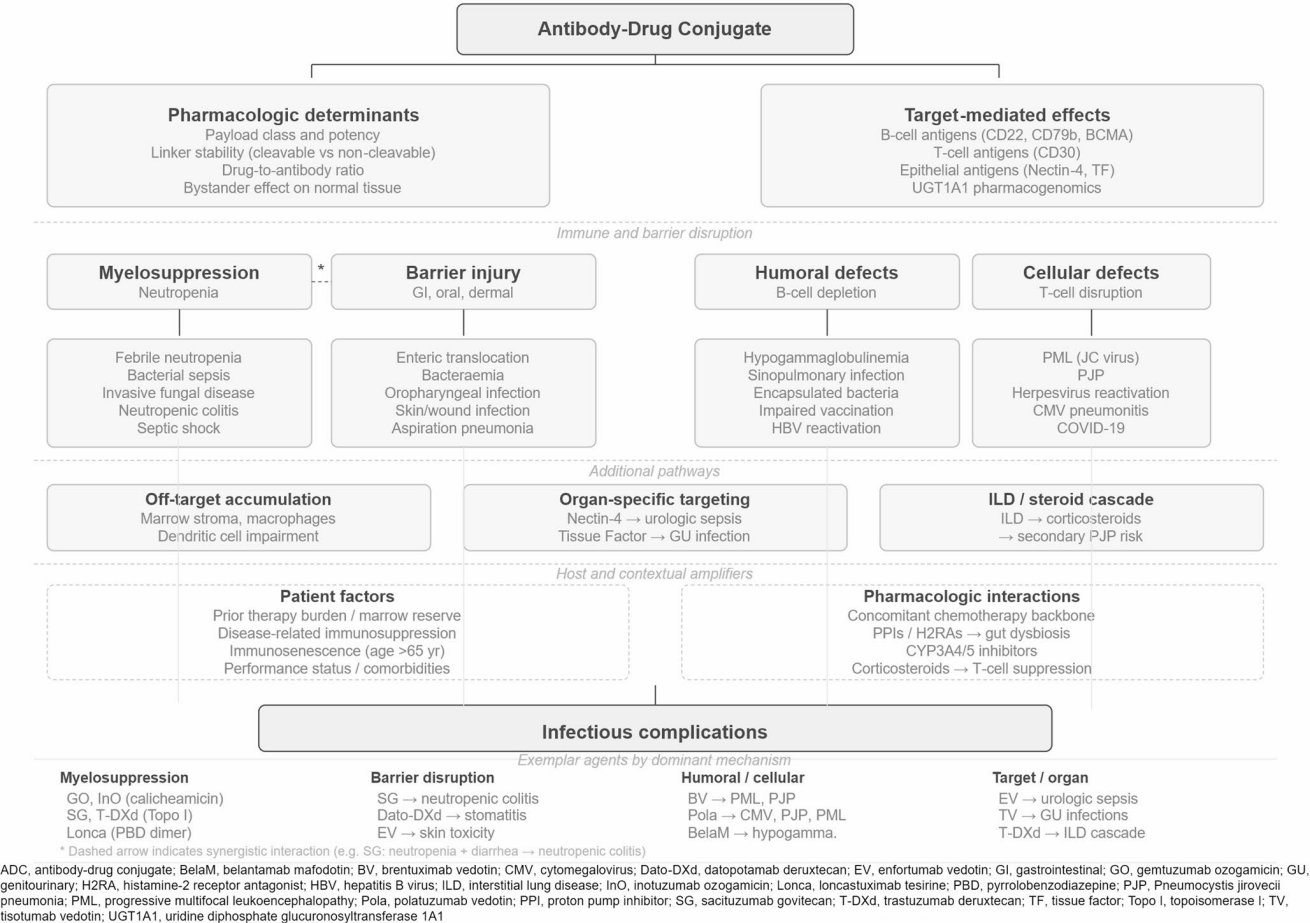
Mechanism of ADC-associated infection risk

Despite the strong overall correlation between neutropenia and infection, several lines of evidence indicate that neutropenia alone does not fully account for the spectrum of ADC-associated infectious risk. The ADC itself may contribute to infectious vulnerability through mechanisms independent of neutrophil count, with several complementary pathways illustrated in Fig. 1.

#### B-Cell Depletion and Humoral Immunodeficiency

Preclinical studies using non-human primate models have demonstrated that ADCs targeting B-cell antigens such as CD79b and CD22 induce B-cell depletion, with preferential elimination of proliferating B cells [66]. Humoral impairment appears more pronounced with ADC-based regimens compared with non-ADC combinations, as reflected by higher rates of immunoglobulin replacement across studies. In a post-hoc analysis, replacement rateswere 8% with BVd (BelaM + bortezomib + dexamethasone) versus 4% with DVd in DREAMM-7, and 18% with BPd (BelaM + pomalidomide + dexamethasone) versus 9% with PVd (pomalidomide + bortezomib + dexamethasone) in DREAMM-8a, with no reported p-values for these comparison.

#### T-Cell–Mediated Immune Surveillance Disruption

BV carries a label warning for PML, likely related to impaired JC virus immunosurveillance in the central nervous system. This effect has been proposed to result from targeting CD30-expressing activated T-cells, although the exact pathophysiology remains incompletely understood [67]. Clinically, post-marketing reports describe cases of PML occurring early, often after a median of three doses and within weeks of treatment initiation, even in patients without prior chemotherapy or profound lymphopenia.These observations support a drug-specific immunologic toxicity rather than a consequence of cumulative immunosuppression [68]. Pharmacovigilance analyses have also identified PJP among the strongest safety signals for BV, indicating susceptibility to OIs [69]. Both BV and Pola carry label warnings for PML, while Pola’s OI spectrum encompasses herpesvirus reactivation, CMV pneumonitis, and PJP, as confirmed by both trial data and real-world studies [68, 70, 71]. The paradox of T-cell–associated infections with Pola, a CD79b-targeting agent, may be explained by the vedotin-mediated bystander effect. Following intracellular release from targeted B cells, the membrane-permeable payload diffuses into the surrounding microenvironment, causing off-target toxicity in neighboring T cells and myeloid progenitors and contributing to lymphopenia and broader immune impairment [72].

#### Mucosal Barrier Injury

Collateral toxicity of cytotoxic payloads on rapidly dividing epithelial cells, especially microtubule inhibitors [73], or bystander effect-mediated disruption of the integrity of mucosal surfaces [74] can create portals for microbial translocation [75]. The gastrointestinal tract is especially vulnerable, and clinically significant mucositis has been reported with ADCs carrying microtubule and topoisomerase I payloads. SG exemplifies this mechanism: the combination of SN-38–driven diarrhea and profound neutropenia create synergistic conditions for enteric bacterial translocation, manifesting as neutropenic colitis in 1.4–2% of patients. Real-world pharmacovigilance data corroborate this risk, demonstrating markedly elevated reporting odds ratios for neutropenic colitis (ROR 188.02, 95% CI 120.09-294.37) and neutropenic sepsis (ROR 46.02, 95% CI 27.15–77.99), with geriatric patients (> 65 years) showing disproportionate vulnerability [76]. Similarly, Dato-Dxd’s high stomatitis burden raises the possibility of oropharyngeal mucosal disruption as an alternative infection pathway despite minimal myelosuppression [77].

#### Organ-Specific Target-Mediated Effects

The anatomic distribution of infections reveals predilections that cannot be fully explained by systemic immunosuppression. Urologic sepsis and UTIs associated with EV in urothelial carcinoma reflect both the primary tumor location and high Nectin-4 expression within urinary epithelium [78, 79]. In the EV-302 trial, Grade 3–4 UTI rates remained comparable between EV plus pembrolizumab (5%) and gemcitabine-platinum chemotherapy (8%), despite dramatically different neutropenia rates (9% vs. 50%), further supporting a target-mediated mechanism [80]. Tisotumab vedotin demonstrates a parallel pattern, with persistent genitourinary infections despite low neutropenia rates (3–3.6%), consistent with Tissue Factor expression in cervical and urinary tract mucosa. Similarly, Trop-2 is highly expressed in salivary glands; this localized expression pattern likely contributes to the distinct profile of stomatitis and oral infections observed with Trop-2-targeted agents, like Dato-Dxd.

### Toward Agent-Specific Risk Stratification

#### Acute Leukemias (CD33, CD22)

Infection prophylaxis in AML patients receiving GO, is guided by well-established societal recommendations that primarily reflect the high baseline infectious risk of this population. In patients treated with combination therapy, the same prophylactic framework as that used for chemotherapy alone is generally applied [81, 82]. The European Conference on Infections in Leukemia-9 (ECIL-9) guidelines recommend the same approach for patients receiving high-dose GO monotherapy in the relapsed setting, whereas no routine antimicrobial prophylaxis is advised for lower-intensity monotherapy [83].

The Prevention and Treatment of Cancer-Related Infections National Comprehensive Cancer Network (NCCN) 2025 guidelines identify bacterial infections, opportunistic fungal infections, and PJP as explicitly recognized risks of CD33-targeted agents, alongside drug-induced VOD, neutropenic colitis, and interstitial pneumonitis [84].

CD22-targeted InO demonstrates a more favorable infectious profile despite use in high-risk ALL population and utilizing an identical to GO calicheamicin payload. InO-treated patients demonstrated lower FN rates than the chemotherapy comparator (26% vs. 53%) and lower grade ≥ 3 infection rates (28% vs. 54%) (15). The ECIL-9 guidelines note that InO monotherapy does not increase the risk of infection beyond the baseline disease state and does not mandate specific antimicrobial prophylaxis [83]. NCCN 2025 guidelines similarly note limited data on specific infections for the CD22 target [84]. This favorable infectious profile degrades when InO is administered in combination regimens, with infection rates rising (73% versus 17% with monotherapy) [85]. Like GO, InO carries a strict Boxed Warning for VOD/SOS, necessitating caution with concomitant hepatotoxic agents.

For both agents, the Hematopoietic Growth Factors National Comprehensive Cancer Network (NCCN) guidelines refer G-CSF use to disease-specific recommendations [86], rather than to the standard FN risk classification applied in non-leukemic malignancies, as discussed below.

#### Multiple Myeloma (BCMA)

Multiple myeloma is fundamentally characterized by severe, progressive immune dysregulation, including profound hypogammaglobulinemia and compromised cellular immunity. This categorizes the disease as an intermediate-to-high risk state for infectious complications within NCCN framework, thereby prompting a “consider prophylaxis” approach across various therapeutic regimens; accordingly, antimicrobial prophylaxis during neutropenia as well as PJP and viral prophylaxis should be considered [84].It should be noted though, that BelaM may confer additional pneumonia risk beyond baseline despite having a relatively modest FAERS signal (ROR 3.05; 95% CI 2.15–4.32). In the DREAMM-7 trial, grade ≥ 3 pneumonia was more frequent with BVd compared with DVd, (16% vs. 4%) despite similar overall infection rates (70 vs 67%). Increased vigilance for pneumonia in BelaM-treated patients may be reasonable, as the intermediate-risk designation may underestimate this agent’s true infectious potential.

#### Lymphomas (CD30, CD79b, CD19)

The lymphoma patient population has traditionally been considered at variable and often underestimated risk for infectious complications. Both CD30-targeted BV and CD79b-targeted Pola have accumulated sufficient evidence to warrant specific prophylactic recommendations.

For CD30-targeted BV, the NCCN 2025 guidelines recommend consideration of CMV monitoring in seropositive patients and PJP and HSV/VZV prophylaxis, based on the drug-induced neutropenia and lymphocytopenia that characterize this agent [84]. The occurrence of PML further necessitates JC virus awareness. Moreover, when BV is used in combination regimens, it carries a high risk for FN, and both NCCN as well as the Infectious Diseases Working Party of the German Society for Hematology and Medical Oncology (AGIHO) 2026 guidelines provide a clear recommendation for primary G-CSF prophylaxis [19, 86, 87].

For CD79b-targeted Pola, the NCCN 2025 guidelines recommend consideration of PJP and HSV/VZV prophylaxis based on concomitant immunosuppression, and highlight the risk of CMV reactivation, PML, fungal infections, and HBVr [84]. The FDA label explicitly recommends PJP and herpesvirus prophylaxis [20]. These data are corroborated by real-world evidence through the pharmacovigilance study analyzing the FAERS database, which identified a highly significant ROR of 8.30 for Pola-induced sepsis [10]. A more recent analysis of the FAERS database reveals a broad spectrum of infectious risks, with significant reporting signals for opportunistic infections (ROR 4.52), COVID-19 (ROR 3.19), and infective pneumonia (ROR 2.54). These infectious complications are likely exacerbated by underlying hematologic toxicities, as evidenced by disproportionately high reporting for agranulocytosis (ROR 12.69), hematopoietic leukopenia (ROR 11.87), and pancytopenia (ROR 10.35) [88]. Collectively, these robust signals make the case for proactive G-CSF administration compelling. Updated NCCN guidelines categorize patients treated with Pola in combination with R-CHP for DLBCL as high risk for FN [86].

CD19-targeted Lonca, induced grade 3–4 neutropenia in 30% with pneumonia in 5% and fatal infection in 1% [21]. In a recent, small international clinician survey (*n* = 15) assessing real-world management of adverse events with Lonca in R/R DLBCL, neutropenia was managed mainly with treatment modifications, with 33.3% of respondents reporting dose delays/holds and 33.3% dose reductions due to myelosuppression [89]. G-CSF use was mentioned but not quantified, and no consistent antimicrobial prophylaxis strategy was reported, indicating a lack of a standardized preventive approach in this setting.

#### Solid Tumors (HER2, Trop-2, Nectin-4, Tissue Factor, c-Met, FRα)

Patients with solid tumors are generally considered at lower baseline infectious risk compared to hematologic populations, and the NCCN guidelines do not provide agent-specific prophylaxis recommendations for ADCs in this setting [84, 86]. Nevertheless, growing evidence supports proactive management for certain agents. SG carries the strongest data: in ASCENT-03, all SG-related deaths were infection-related, occurring predominantly in early cycles among patients without G-CSF prophylaxis who had additional FN risk factors [90]. The NCCN guidelines accordingly classify SG-treated patients at intermediate FN risk (10–20%), with prophylaxis guided by patient-specific factors [86]. Both real-world and prospective data support G-CSF use, with a nationwide analysis of 381 SG-treated patients demonstrating lower neutropenia rates with prophylactic G-CSF (25% versus 44%) [91] and a phase 2 study showing that primary prophylaxis reduced grade ≥ 3 neutropenia from 40% to 16% (*p* = 0.0002) with no FN events [92]. Neutropenia with SG is front-loaded, with a median onset of 12 days that attenuates over time [93], though optimal G-CSF timing remains unclear; short-acting formulations on days 4–6 or pegfilgrastim 24–48 h post-infusion have been proposed [94]. Real-world practice remains variable, with primary prophylaxis used in 40% of patients but ultimately required in up to 79% [95].

These observations should be interpreted within the broader context of ADC pharmacokinetics. A scoping review by the Neutropenic Events Working Group emphasizes that ADC-associated neutropenia management remains insufficiently standardized, and G-CSF prophylaxis should be individualized based on treatment characteristics and patient-specific risk factors [96]. Furthermore, UGT1A1 genotyping may predict SG-associated neutropenia risk, as UGT1A1 variant carriers demonstrated significantly higher FN incidence (33.3% vs. 6.7%; *p* = 0.025) in a recent real-world study [97], although current guidelines do not recommend preemptive dose modification based on genotype [98].

SG is the only solid tumor ADC listed in NCCN FN risk classification tables; the absence of other agents reflects a lack of formal risk stratification rather than confirmed low risk. Beyond FN, additional agent-specific infection considerations warrant attention. T-DXd-induced ILD frequently necessitates prolonged corticosteroid treatment, rendering patients susceptible to opportunistic pulmonary infections including PJP. Dato-DXd is characterized by high rates of stomatitis (71% any grade), for which proactive oral hygiene education and prophylactic steroid-containing mouthwashes have been proposed to maintain mucosal integrity [99]. For EV, urologic complications warrant a low threshold for urine cultures in symptomatic patients, while recent FAERS data have identified a previously unreported association with oral candidiasis during neutropenic episodes [100]. Finally, tisotumab vedotin and telisotuzumab vedotin present uncommon but clinically consequential infection signals—sepsis in 2–3% and fatal pneumonia in 1.2%, respectively—warranting awareness during treatment monitoring.

#### Emerging Signals for Protective Interventions

The results reported by Xia et al. in their comprehensive pharmacovigilance study should also inform current practices. Firstly, approximately 60% of ADC-associated sepsis events occur within the first month of therapy [10]. This should prompt concentrating maximum prophylactic intensity, during the initial treatment cycles, rather than adopting a reactive approach. Co-administration of ADCs with common proton pump inhibitors (PPIs), H2-receptor antagonists and CYP3A4/5 strong inhibitors significantly increased the risk of severe sepsis. The risk for septic shock is particularly elevated when ADCs are combined with CYP3A4 inhibitors (ROR 13.91). Interestingly, G-CSF co-administration did not attenuate the sepsis signal. This finding does not contradict the established clinical benefit of G-CSF in reducing FN but further underscores the uncertainty regarding the optimal use of G-CSF in ADC-treated patients and highlights that growth factor support alone incompletely addresses the multifactorial infectious risk of ADC-based treatments as described throughout this review.

### Limitations

This analysis has several limitations. Data were extracted from FDA prescribing information, which represents a curated summary of pivotal trial safety data rather than comprehensive adverse event ascertainment. Post-marketing surveillance captures events that may not have been systematically collected in trials but are subject to reporting bias and cannot establish causality. Cross-trial comparisons are inherently limited by differences in patient populations, prior therapy exposure, combination regimens, and adverse event reporting methodologies. Furthermore, many ADCs have received approval based on relatively small registration trials in heavily pretreated populations, which may not reflect safety profiles in broader clinical use. The BelaM discordance, in particular, may partially reflect limited FAERS data accumulation due to recent approval and restricted distribution. Finally, the COVID-19 pandemic introduced a confounding variable in infection reporting across trials conducted during 2020–2023, complicating interpretation of respiratory infection rates for several agents.

## Conclusion

Future investigations should elucidate the mechanistic basis for differential infectious risk, examining contributions of payload selection and chemistry, linker technology, antibody engineering and modifications, dosing strategies, and target expression patterns on normal cells [101–107]. Such insights could inform next-generation ADC design to minimize infectious complications while maintaining therapeutic efficacy. Equally important, prospective studies specifically designed to capture infection outcomes, rather than relying on adverse event reporting within efficacy-oriented trials, are needed to accurately characterize the true burden of ADC-associated infections in real-world clinical practice. As the ADC landscape continues to expand, with over a hundred agents currently in clinical development [108], the framework presented here provides a basis for evidence-based infection prevention.

## Data Availability

No datasets were generated or analysed during the current study.

## Key References

- Zhu Y, Liu K, Wang K, Zhu H. Treatment-related adverse events of antibody-drug conjugates in clinical trials: A systematic review and meta-analysis. Cancer. 2023;129:283–295.

- ○ This large-scale meta-analysis provides the most comprehensive quantification to date of ADC-related toxicities, establishing that 91.2% of patients experience at least one treatment-related adverse event and that pneumonia and sepsis are leading causes of treatment-related death.

- Tang SC, Wynn C, Le T, et al. Influence of antibody-drug conjugate cleavability, drug-to-antibody ratio, and free payload concentration on systemic toxicities: A systematic review and meta-analysis. Cancer Metastasis Rev. 2024;44:18.

- ○ This meta-analysis is the first to quantify the differential toxicity impact of cleavable versus non-cleavable ADC linkers, demonstrating a significantly higher rate of grade ≥ 3 adverse events with cleavable constructs (weighted risk difference − 12.9%), with neutropenia accounting for the largest absolute difference.

- Tan HN, Morcillo MA, Lopez J, et al. Treatment-related adverse events of antibody drug-conjugates in clinical trials. J Hematol Oncol. 2025;18:71.

- ○ This systematic review identified a drug-to-antibody ratio exceeding 4 as an independent predictor of hematologic toxicity, associated with an 18.3-fold increase in odds.

- Baden LR, Swaminathan S, Almyroudis NG, et al. Prevention and Treatment of Cancer-Related Infections, Version 3.2024, NCCN Clinical Practice Guidelines in Oncology. J Natl Compr Canc Netw. 2024;22:617–644.

- ○ This updated NCCN guideline provides the most current evidence-based framework for infection prevention and treatment in oncology patients. It serves as the primary reference for the prophylaxis recommendations discussed in the clinical management section of this review.

- Sandherr M, Schalk E, Heinz WJ, et al. Evidence-based AGIHO guideline update on prophylaxis of infectious complications with granulocyte-stimulating factors (G-CSF) for the treatment of adult patients with cancer. Eur J Cancer. 2026;235:116244.

- ○ The latest AGIHO G-CSF guideline update consolidates evidence for granulocyte colony-stimulating factor prophylaxis in adult cancer patients treated with myelosuppressive regimens, including ADC-containing combinations.

## Abbreviation

ABVD: Adriamycin, bleomycin, vinblastine, dacarbazine.
ADC: Antibody–drug conjugate.
AGIHO: Infectious diseases working party of the german society for hematology and medical oncology.
ALCL: Anaplastic large cell lymphoma.
ALL: Acute lymphoblastic leukemia.
AML: Acute myeloid leukemia.
ANC: Absolute neutrophil count.
ASCT: Autologous stem cell transplantation.
AVD: Adriamycin, vinblastine, dacarbazine.
BBW: Black Box Warning.
BC: Breast cancer.
BCMA: B-cell maturation antigen.
BelaM: Belantamab mafodotin.
BPd: Belantamab mafodotin + pomalidomide + dexamethasone.
BR: Bendamustine + rituximab.
BSC: Best supportive care.
BV: Brentuximab vedotin.
BVd: Belantamab mafodotin + bortezomib + dexamethasone.
CAR-T: Chimeric antigen receptor T-cell.
CI: Confidence interval.
CLS: Capillary leak syndrome.
CMV: Cytomegalovirus.
COVID-19: Coronavirus disease 2019.
CTCAE: Common terminology criteria for adverse events.
CYP3A4: Cytochrome P450 3A4.
DAR: Drug-to-antibody ratio.
Dato-DXd: Datopotamab deruxtecan.
DDI: Drug–drug interaction.
DLBCL: Diffuse large B-cell lymphoma.
DVd: Daratumumab + bortezomib + dexamethasone.
DXd: Deruxtecan.
EBMT: European society for blood and marrow transplantation.
ECIL: European conference on infections in leukemia.
EHA: European hematology association.
EV: Enfortumab vedotin.
FAERS: FDA adverse event reporting system.
FDA: United States food and drug administration.
FN: Febrile neutropenia.
FQ: Fluoroquinolone.
FRα: Folate receptor alpha.
FT: Fallopian tube.
G-CSF: Granulocyte colony-stimulating factor.
GI: Gastrointestinal.
GO: Gemtuzumab ozogamicin.
HBV: Hepatitis B virus.
HCL: Hairy cell leukemia.
HCV: Hepatitis C virus.
HER2: Human epidermal growth factor receptor 2.
HGBL: High-grade B-cell lymphoma.
HIV: Human immunodeficiency virus.
HL: Hodgkin lymphoma.
HR: Hormone receptor.
HSCT: Hematopoietic stem cell transplantation.
HSV: Herpes simplex virus.
HUS: Hemolytic uremic syndrome.
IDSA: Infectious diseases society of America.
IFD: Invasive fungal disease.
ILD: Interstitial lung disease.
IL-6: Interleukin-6.
InO: Inotuzumab ozogamicin.
IPI: International prognostic index.
JC virus: John Cunningham virus.
JCV: John Cunningham virus.
LA/M: Locally advanced or metastatic.
LBCL: Large B-cell lymphoma.
LFTs: Liver function tests.
Lonca: Loncastuximab tesirine.
mBC: Metastatic breast cancer.
MDS: Myelodysplastic syndrome.
MM: Multiple myeloma.
MMAE: Monomethyl auristatin E.
MMAF: Monomethyl auristatin F.
MRSA: Methicillin-resistant Staphylococcus aureus.
mTNBC: Metastatic triple-negative breast cancer.
NCCN: National comprehensive cancer network.
NSCLC: Non-small cell lung cancer.
OR: Odds ratio.
PBD: Pyrrolobenzodiazepine.
PD-1/PD-L1: Programmed death-1/programmed death-ligand 1.
PJP: Pneumocystis jirovecii pneumonia.
PML: Progressive multifocal leukoencephalopathy.
PNA: Purine nucleoside analog.
Pola: Polatuzumab vedotin.
PPIs: Proton pump inhibitors.
PTCL: Peripheral T-cell lymphoma.
PVd: Pomalidomide + bortezomib + dexamethasone.
R-CHOP: Rituximab, cyclophosphamide, doxorubicin, vincristine, prednisone.
R-CHP: Rituximab, cyclophosphamide, doxorubicin, prednisone.
R/M: Recurrent or metastatic.
R/R: Relapsed or refractory.
ROR: Reporting odds ratio.
RTI: Respiratory tract infection.
sALCL: Systemic anaplastic large cell lymphoma.
SG: Sacituzumab govitecan.
SN-38: Active metabolite of irinotecan (7-ethyl-10-hydroxycamptothecin).
SOS: Sinusoidal obstruction syndrome.
TB: Tuberculosis.
T-DM1: Ado-trastuzumab emtansine (trastuzumab emtansine).
T-DXd: Trastuzumab deruxtecan.
TMP-SMX: Trimethoprim-sulfamethoxazole.
TNBC: Triple-negative breast cancer.
TPC: Treatment of physician’s choice.
Trop-2: Trophoblast cell surface antigen 2.
UC: Urothelial cancer/carcinoma.
UGT1A1: Uridine diphosphate glucuronosyltransferase 1A1.
URTI: Upper respiratory tract infection.
UTI: Urinary tract infection.
VOD: Veno-occlusive disease.
VZV: Varicella-zoster virus.
WT: Wild-type.
